# Perinatal mood and anxiety diagnoses and treatment increased among medicaid births, 2017–2021

**DOI:** 10.1038/s41598-026-57879-3

**Published:** 2026-06-18

**Authors:** Kara Zivin, Andrea Pangori, Anca Tilea, Anna Courant, Erin Miller, Stephanie V. Hall, Vanessa K. Dalton, Karen M. Tabb

**Affiliations:** 1 Department of Psychiatry, Michigan Medicine, 2800 Plymouth Road, Building 16, Ann Arbor, MI 48109 USA; 2 Department of Obstetrics and Gynecology, Michigan Medicine, Ann Arbor, MI USA; 3 Center for Clinical Management Research, VA Ann Arbor Healthcare System, Ann Arbor, MI USA; 4 School of Social Work, University of Illinois at Urbana-Champaign, Urbana, IL USA

**Keywords:** Medicaid, Perinatal mood and anxiety disorders, Births, Maps, Trends, Diagnosis, Psychology

## Abstract

Despite funding the most births in the United States (US), we know little about basic patterns and trends of mental health conditions among Medicaid-insured women during pregnancy and after delivery. We sought to characterize the prevalence of diagnosis and treatment for perinatal mood and anxiety disorders (PMAD) among women with Medicaid health insurance coverage. We conducted serial, cross-sectional analyses of women with live-birth deliveries during 2017–2021 using Transformed Medicaid Statistical Information System (T-MSIS) claims. To construct state-specific PMAD diagnosis and treatment trajectories including antidepressant prescriptions and psychotherapy visits, we examined geographic variations by state and year among reproductive aged women. In a national cohort of 4,579,289 women with Medicaid and a live birth, 635,785 (13.88%) had a PMAD diagnosis during the study period. Perinatal women in all age, race, ethnicity, geographic regions, comorbidity burdens, and all but one state experienced growth in PMAD diagnoses. Substantial variation occurred among states for antidepressant use, while national trends remained stable. Characterizing PMAD diagnosis and treatment patterns represents an important public health contribution. Future research should aim to better understand factors underlying variation in PMAD diagnoses and treatment across subgroups and geographic areas.

**Supplementary Information:**

The online version contains supplementary material available at https://doi.org/10.1038/s41598-026-57879-3.

## Introduction

More than a decade after researchers declared that there is “no health without perinatal mental health^1–3^,” the global burden of perinatal mental health conditions such as depression and anxiety remains high, with up to one in five women affected and higher rates in low- to middle-income countries compared to high-income countries^4^. Awareness of such mental health conditions (also known as perinatal mood and anxiety disorders or PMAD) in the United States (US) has increased over time^5,6^. Yet these mental health conditions remain common during pregnancy and after birth in the US^7,8^, with few women receiving treatment and fewer achieving remission^9^. Potential negative consequences for women and infants extend from decreased quality of life^10,11^ to worsening maternal mental health (ranging in severity to perinatal suicidality^12^, and adverse infant outcomes, such as preterm birth, low birthweight, and negative childhood health impacts^13–16^. Additionally, these negative intergenerational effects expand further to partners and families^17,18^. Maternal mental health conditions represent a leading cause of maternal morbidity and mortality^19^, and the US has the highest rate of maternal mortality in the developed world^20^.

Over the last two decades, multiple state and federal laws and guidelines have aided efforts to improve PMAD detection, diagnosis, and treatment^21^. Our team documented the prevalence of these outcomes among privately insured individuals between 2008 and 2020, highlighting increases in both diagnosis and treatment over time across the population, and within patient subgroups and geographic locations^5,22,23^. Despite progress in perinatal mental health assessment, basic comprehensive data on the national prevalence of PMAD diagnoses and treatment within Medicaid, the largest single insurer of U.S. births, remains lacking. Previous studies reflect older data or merely focus on a single state^24–26^.

As with any population-level health condition, patterns of diagnosis and treatment likely vary over time in response to changes in clinical awareness, diagnostic criteria, service provision, and external shocks, such as the pandemic^27^. However, without comprehensive analysis, the medical and research communities cannot determine how diagnosis and treatment have changed. To address this gap in the scientific literature, we sought to calculate the prevalence of PMAD diagnoses in women with Medicaid insurance as well as psychotherapy and/or antidepressant medication treatment among these women with PMAD diagnoses. In addition, we sought to characterize both annual rates and changes over time in the prevalence of diagnosis and treatment for PMAD both within and across the United States. We hypothesized that diagnosis and treatment would increase over time.

## Methods

### Study design

We conducted serial, cross-sectional analyses of women with live-birth deliveries during 2017–2021 using deidentified claims from the Transformed Medicaid Statistical Information System (T-MSIS) Analytic Files, known as TAF^28^. This data source covers Medicaid-insured women across all fifty U.S. states and Washington, D.C. To construct state-specific PMAD diagnosis and treatment trajectories, we examined geographic variations by state and year among women with deliveries resulting in a live birth.

We conducted this study in accordance with the Declaration of Helsinki and received approval from the University of Michigan Institutional Review Board (HUM00204182) as an exempt study due to the project analyzing only pre-existing data. IRB approval encompassed the study protocol, a waiver of informed consent, and a waiver of HIPAA authorization for the entire project.

### Data source

The T-MSIS Analytic Files (TAF) are administrative claims data compiled by the Centers for Medicare and Medicaid Services from state Medicaid programs. Medicaid finances approximately 43% of all US births, with state-level variation in coverage rates reflecting differences in eligibility thresholds. Medicaid covers individuals who qualify based on income, pregnancy, disability, or categorical eligibility; pregnant women typically qualify at income levels up to 138–200% of the federal poverty level, depending on state^29^. TAF includes closed claims for inpatient hospitalizations, outpatient visits, emergency department encounters, and prescription drug dispensing at pharmacies; it does not capture medications administered in hospital settings. Data completeness varies across states and years. A claims-processing lag of approximately 12–18 months means that the most recent complete year available at the time of analysis was 2021. Medicaid benefits vary across states, including variation in covered services for prenatal care and mental health; this variation is a potential source of heterogeneity in diagnosis and treatment rates that we discuss in the Limitations section.

### Participants

Our cohort of deliveries included women aged 15–44, based on the Center for Disease Control’s (CDC) definition of reproductive age^30^, with a live birth (excluding abortion, dilatation and curettage, and stillbirth) and continuous enrollment for nine months before through three months after delivery^30^. Continuous enrollment means the beneficiary was enrolled in Medicaid for every month of our observation period (nine months before through three months after delivery without losing insurance. We chose this 12-month time window a-priori based on our prior work to include the typical nine month pregnancy timeline because many individuals become eligible for Medicaid due to pregnancy, and they could also lose coverage 60 days after delivery^31^.

To code deliveries, we employed International Classification of Diseases, Clinical Modification (ICD-10-CM), diagnoses and procedures, and Current Procedural Terminology (CPT) codes. All diagnosis, procedure, and prescription codes used in this study appear in Supplementary Table S1. Within each year, about 99.97% of deliveries represent unique individuals, with 2021 having 100% unique individuals.

### Primary outcomes

This study included three outcomes, which we assessed during the 12 months surrounding the delivery. First, *PMAD diagnosis*, we used the Healthcare Cost and Utilization Project algorithm^32,33^, which requires one inpatient claim or two outpatient claims with a PMAD diagnosis code on different service dates (i.e., distinct calendar dates, with no minimum interval beyond that). Second, *antidepressant prescriptions*, defined as at least one prescription for any dosage of antidepressant using Hierarchical Ingredient Codes^31^. Finally, *psychotherapy use*, defined as at least one claim with CPT codes for psychotherapy visits^34^. We calculated the PMAD rate out of all deliveries and calculated antidepressant and psychotherapy rates among all deliveries with PMAD. All diagnosis, prescription, and procedure codes appear in Supplementary Table S1.

### Beneficiary demographic and clinical characteristics

We included patient-level demographic characteristics, namely age (15–19, 20–24, 25–29, 30–35, and 36–44), race and ethnicity (American Indian and Alaska Native (AIAN) non-Hispanic; Asian non-Hispanic; Black non-Hispanic; Hawaiian/Pacific Islander; Hispanic all races; Multiracial non-Hispanic; Other Non-Hispanic; and White non-Hispanic), and comorbid medical conditions (0–1, 2+).

To capture comorbid medical conditions, we used the Bateman Obstetric Comorbidity Index (OBCMI), a validated risk score for severe maternal morbidity that incorporates comorbid diagnoses that increase risk for adverse outcomes, calculated during pregancy^35^. Scores range from 0 to 45. Higher scores reflect more medically complex patients at greater risk for complications or poor outcomes. OBCMI scores skew right, with scores that exceed 15 and a median score of 2, therefore, we dichotomized the score as 0–1 or 2 or higher to reflect the median index score^36^. Finally, to classify geographic information, we grouped states into four regions based on U.S. Census Bureau regional divisions (Midwest, Northeast, South, and West)^37,38^.

### Data analysis

With deliveries as our unit of analysis, we summarized demographic characteristics for all deliveries and those with PMAD overall and by year (2017–2021). We calculated PMAD diagnosis per 10,000 deliveries ((# of deliveries with PMAD/# of deliveries) * 10,000) by age, race and ethnicity, region, and OBCMI score for each year and created a range plot with the first and last years of the study period (2017 and 2021, respectively). When we summarized race and ethnicity counts or calculated PMAD diagnosis by race and ethnicity, we only included states that had data quality of medium or low concern defined by Medicaid’s Data Quality Atlas^39^. (See Supplement Table S3 for included states by year). We then calculated PMAD diagnoses per 10,000 deliveries by state and year from 2017 to 2021 as well as antidepressant prescriptions and psychotherapy use among deliveries with PMAD diagnoses (i.e. (# of deliveries with Antidepressant amongst those with PMAD / # of deliveries with PMAD) * 10,000). We generated range plots, with the first and last years of the study period to anchor the start and end for each state.

We used state-specific linear regressions to identify annual outcome changes per 10,000 deliveries with choropleth maps shaded to visualize values over geographical areas. We categorized the choropleth maps’ color categories using quantiles, unless a quantile crossed over from a negative to positive change; then we adjusted those categories accordingly. Finally, we used linear regression to quantify the annual outcome changes per 10,000 deliveries for each region. Due to small counts for Mississippi in 2021, we removed Mississippi from the state-specific range plot and used only years 2017 through 2020 for the annual change models and maps.

## Results

In a national cohort of 4,579,289 deliveries with Medicaid health insurance coverage [for the 9 months before and 3 months after delivery] and a live birth between 2017 and 2021, 635,785 (13.88%) had a PMAD diagnosis during the perinatal period (Table 1). Whereas the overall size of the delivery population grew from 964,996 in 2017 to 983,254 in 2021, an 89% relative increase (0.4% absolute increase), PMAD diagnoses increased from 113,851 in 2017 to 148,415 in 2021, a 30.36% relative increase (5.4% absolute increase) over a five-year period.

**Table 1:** Sociodemographic characteristics of U.S. Medicaid insured delivering women with a perinatal mood and anxiety disorder (PMAD) diagnosis, 2017–2021.

	Deliveries (9 months enrollment before, 3 months enrollment after)						PMAD among delivery cohort					
	2017–2021	2017	2018	2019	2020	2021	2017–2021	2017	2018	2019	2020	2021
	**4**,**579**,**289**	**964**,**996**	**940**,**041**	**893**,**920**	**797**,**078**	**983**,**254**	**635**,**785**	**113**,**851**	**121**,**356**	**127**,**219**	**124**,**944**	**148**,**415**
PMAD	635,785 (13.88)	113,851 (11.80)	121,356 (12.91)	127,219 (14.23)	124,944 (15.68)	148,415 (15.09)	--	--	--	--	--	--
Antidepressant	626,648 (13.68)	116,136 (12.03)	124,046 (13.20)	127,617 (14.28)	118,773 (14.9)	140,076 (14.25)	292,207 (45.96)	50,680 (44.51)	55,956 (46.11)	59,886 (47.07)	59,038 (47.25)	66,647 (44.91)
Psychotherapy	802,609 (17.53)	156,140 (16.18)	168,173 (17.89)	176,194 (19.71)	146,514 (18.38)	155,588 (15.82)	309,131 (48.62)	55,343 (48.61)	60,188 (49.6)	64,858 (50.98)	61,298 (49.06)	67,444 (45.44)
Age Group												
15–19	350,964 (7.66)	82,363 (8.54)	76,065 (8.09)	69,499 (7.77)	58,582 (7.35)	64,455 (6.56)	47,036 (7.4)	9,431 (8.28)	9,624 (7.93)	9,756 (7.67)	8,979 (7.19)	9,246 (6.23)
20–24	1,258,430 (27.48)	277,750 (28.78)	262,501 (27.92)	243,109 (27.2)	212,092 (26.61)	262,978 (26.75)	162,001 (25.48)	29,217 (25.66)	30,667 (25.27)	32,175 (25.29)	31,462 (25.18)	38,480 (25.93)
25–29	1,413,038 (30.86)	301,990 (31.29)	294,383 (31.32)	277,338 (31.02)	241,772 (30.33)	297,555 (30.26)	196,319 (30.88)	35,788 (31.43)	38,165 (31.45)	39,145 (30.77)	37,812 (30.26)	45,409 (30.6)
30–35	1,106,193 (24.16)	216,105 (22.39)	218,406 (23.23)	215,417 (24.1)	201,577 (25.29)	254,688 (25.9)	163,761 (25.76)	28,124 (24.7)	30,455 (25.1)	32,580 (25.61)	33,096 (26.49)	39,506 (26.62)
36–44	450,664 (9.84)	86,788 (8.99)	88,686 (9.43)	88,557 (9.91)	83,055 (10.42)	103,578 (10.53)	66,668 (10.49)	11,291 (9.92)	12,445 (10.25)	13,563 (10.66)	13,595 (10.88)	15,774 (10.63)
Region												
***Missing***	200 (< 0.01)	42 (< 0.01)	--	137 (0.01)	--	--	--	--	--	22 (< 0.01)	--	--
Midwest	973,688 (21.26)	213,749 (22.15)	202,967 (21.59)	187,448 (20.97)	167,129 (20.97)	202,395 (20.58)	171,670 (27.)	32,696 (28.72)	33,503 (27.61)	34,199 (26.88)	33,021 (26.43)	38,251 (25.77)
Northeast	737,721 (16.11)	155,695 (16.13)	153,187 (16.3)	144,857 (16.2)	126,281 (15.84)	157,701 (16.04)	125,274 (19.7)	23,727 (20.84)	24,728 (20.38)	24,852 (19.53)	23,568 (18.86)	28,399 (19.13)
South	1,696,796 (37.05)	335,458 (34.76)	336,799 (35.83)	330,328 (36.95)	302,304 (37.93)	391,907 (39.86)	202,634 (31.87)	32,880 (28.88)	37,021 (30.51)	40,146 (31.56)	41,284 (33.04)	51,303 (34.57)
West	1,170,884 (25.57)	260,052 (26.95)	247,081 (26.28)	231,150 (25.86)	201,357 (25.26)	231,244 (23.52)	136,174 (21.42)	24,540 (21.55)	26,104 (21.51)	28,000 (22.01)	27,069 (21.66)	30,461 (20.52)
OBCMI												
**< 2**	3,801,591 (83.02)	815,523 (84.51)	787,572 (83.78)	732,297 (81.92)	644,282 (80.83)	821,917 (83.59)	466,792 (73.42)	84,585 (74.29)	89,013 (73.35)	91,825 (72.18)	89,518 (71.65)	111,851 (75.36)
**≥ 2**	777,698 (16.98)	149,473 (15.49)	152,469 (16.22)	161,623 (18.08)	152,796 (19.17)	161,337 (16.41)	168,993 (26.58)	29,266 (25.71)	32,343 (26.65)	35,394 (27.82)	35,426 (28.35)	36,564 (24.64)

	2017–2021	2017	2018	2019	2020	2021	2017–2021	2017	2018	2019	2020	2021
	3,144,119	634,659	610,313	598,521	543,743	756,883	426,052	72,597	75,937	82,492	82,856	112,170
Race/Ethnicity^§^												
*Missing*	49,045 (1.56)	11,942 (1.88)	10,723 (1.76)	6,932 (1.16)	9,185 (1.69)	10,263 (1.36)	6,045 (1.42)	1,148 (1.58)	1,211 (1.59)	948 (1.15)	1,325 (1.60)	1,413 (1.26)
American Indian/Alaskan Native*	52,222 (1.66)	10,931 (1.72)	10,438 (1.71)	10,362 (1.73)	8,043 (1.48)	12,448 (1.64)	8,314 (1.95)	1,481 (2.04)	1,465 (1.93)	1,747 (2.12)	1,526 (1.84)	2,095 (1.87)
Asian*	79,686 (2.53)	17,587 (2.77)	16,403 (2.69)	14,567 (2.43)	13,221 (2.43)	17,908 (2.37)	3,750 (0.88)	626 (0.86)	701 (0.92)	700 (0.85)	718 (0.87)	1,005 (0.90)
Black*	800,761 (25.47)	155,145 (24.45)	155,940 (25.55)	159,530 (26.65)	138,387 (25.45)	191,759 (25.34)	83,453 (19.59)	13,501 (18.60)	15,338 (20.20)	16,902 (20.49)	16,254 (19.62)	21,458 (19.13)
Hawaiian/Pacific Islander*	16,849 (0.54)	3,761 (0.59)	3,620 (0.59)	3,441 (0.57)	2,695 (0.50)	3,332 (0.44)	1,032 (0.24)	209 (0.29)	179 (0.24)	196 (0.24)	206 (0.25)	242 (0.22)
Hispanic	802,128 (25.51)	165,277 (26.04)	156,225 (25.60)	150,084 (25.08)	137,046 (25.20)	193,496 (25.56)	65,936 (15.48)	11,163 (15.38)	11,306 (14.89)	12,712 (15.41)	12,584 (15.19)	18,171 (16.20)
Multiracial*	18,687 (0.59)	2,645 (0.42)	2,575 (0.42)	2,525 (0.42)	3,031 (0.56)	7,911 (1.05)	3,152 (0.74)	349 (0.48)	376 (0.50)	407 (0.49)	552 (0.67)	1,468 (1.31)
Other*	101,580 (3.23)	24,817 (3.91)	20,636 (3.38)	17,874 (2.99)	15,295 (2.81)	22,958 (3.03)	12,422 (2.92)	2,713 (3.74)	2,460 (3.24)	2,225 (2.70)	1,996 (2.41)	3,028 (2.70)
White*	1,223,161 (38.90)	242,554 (38.22)	233,753 (38.30)	233,206 (38.96)	216,840 (39.88)	296,808 (39.21)	241,948 (56.79)	41,407 (57.04)	42,901 (56.50)	46,655 (56.56)	47,695 (57.56)	63,290 (56.42)

Cells are presented as n(%).

^§^Race and Ethnicity is only reported for states that have medium to low concern race and ethnicity data quality. See Supplement Table S3 for a list of states included each year.

* = non-Hispanic.

**Fig. 1:**
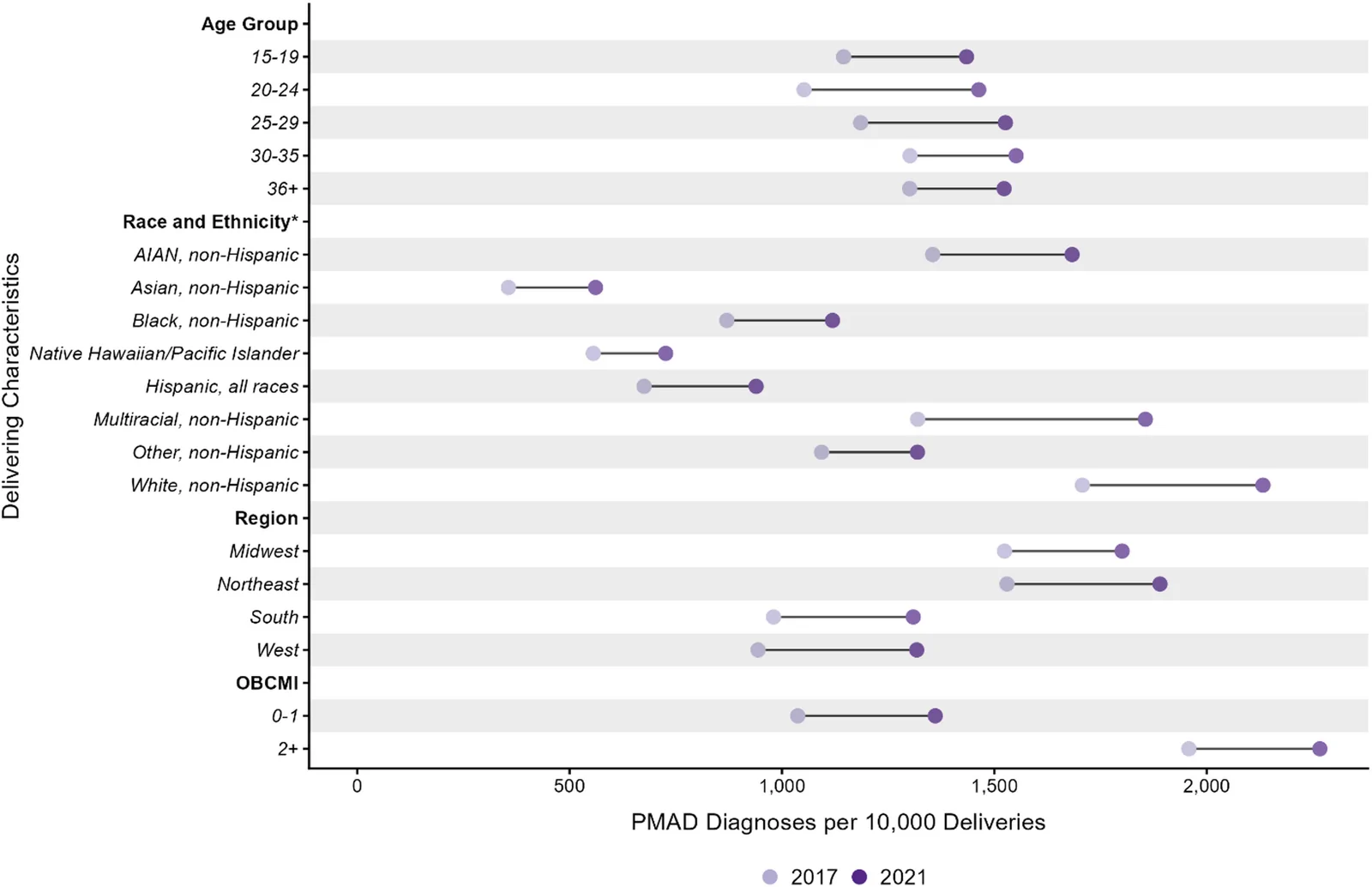
Prevalence of perinatal mood and anxiety disorder (PMAD) per 10,000 deliveries in the U.S. among Medicaid insured women, by age, race and ethnicity, geographic region, and comorbidity status, 2017–2021. *Race and Ethnicity only reported for states with medium to low concern race and ethnicity data quality (See Supplementary Table S3 for list of states included each year).

As shown in Fig. 1, perinatal women in all age, race, ethnicity, geographic, and OBCMI categories experienced growth in PMAD diagnoses over the observation period. Among races and ethnicities, Multiracial, non-Hispanic women experienced the largest increase (40.64% relative increase, 5.36% absolute increase), from 1,319 (SE: 65.8) in 2017 to 1,856 (SE: 43.7) per 10,000 deliveries in 2021. In 2017,the initial prevalence of PMAD diagnoses were highest among older women (ages 30–44), yet younger women (ages 20–24) experienced a larger increase in diagnoses (39.10% relative increase, 4.11% absolute increase), from 1,052 (SE: 5.8) in 2017 to 1,463 (SE: 6.9) per 10,000 deliveries in 2021. Figure 1 values and standard errors appear in Supplementary Table S2.

Among geographic regions, the Midwest and Northeast had higher PMAD diagnoses in both 2017 and 2021 than the South and West. The West saw the largest increase in diagnoses (39.59% relative increase; 3.74% absolute increase), from 944 (SE: 5.1) to 1,317 (SE: 7.0) per 10,000 deliveries. Although those with more comorbidities (OBCMI 2+) had higher PMAD diagnoses, those with fewer comorbidities (OBCMI 0–1) experienced a slightly greater increase in PMAD diagnosis (31.21% relative increase; 3.24% absolute increase), from 1,037 (SE: 3.4) in 2017 to 1,361 (SE: 3.8) in 2021 per 10,000 deliveries compared to those with more comorbidities (OBCMI 2+) (15.75% relative increase; 3.08% absolute increase), from 1,958 (SE: 10.3) to 2,266 (SE: 10.4) per 10,000 deliveries.

**Fig. 2:**
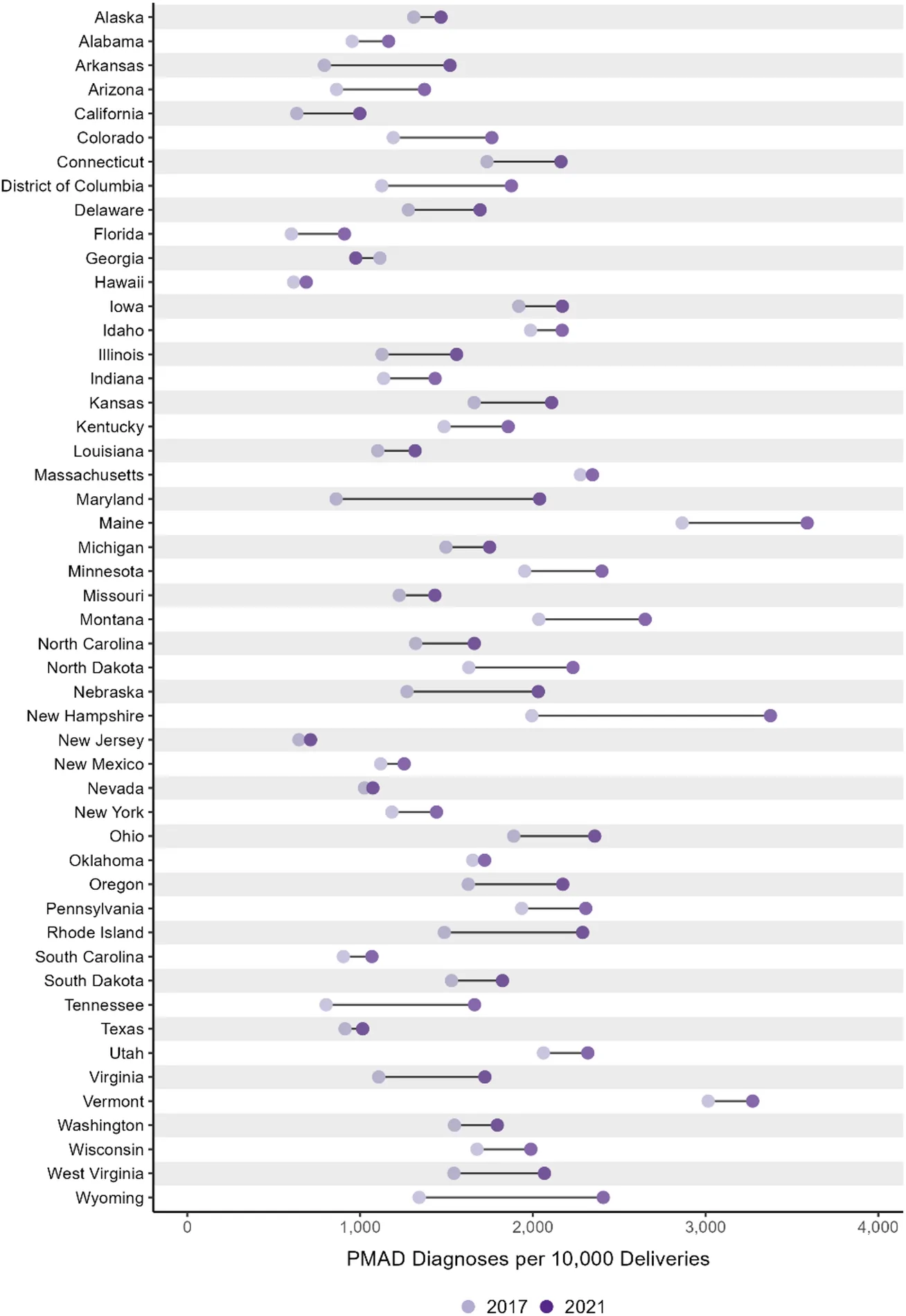
Prevalence of perinatal mood and anxiety disorder (PMAD) per 10,000 deliveries in the U.S. among Medicaid insured women by state, 2017–2021. *Mississippi is removed from this figure due to small counts in 2021.

Figure 2 shows that all states except Georgia experienced increasing rates of PMAD diagnoses between 2017 and 2021. Florida (602 (SE: 8.9) per 10,000 deliveries), Hawaii (615 (SE: 34.2) per 10,000 deliveries), California (633 (SE: 6.8) per 10,000 deliveries), and New Jersey (646 (SE: 19.4) per 10,000 deliveries) experienced the lowest PMAD diagnosis rates per 10,000 deliveries in 2017. Vermont (3,015 (SE: 105.1) per 10,000 deliveries), Maine (2,865 (SE: 86.9) per 10,000 deliveries), and Massachusetts (2,276 (SE: 30.9) per 10,000 deliveries) experienced the highest PMAD diagnosis rates per 10,000 deliveries in 2017. Similar to 2017, in 2021, Hawaii (688 (SE: 40.4) per 10,000 deliveries), New Jersey (713 (SE: 18.8) per 10,000 deliveries), and Florida (909 (SE: 10.5) per 10,000 deliveries) experienced the lowest PMAD diagnosis rates. Maine (3,589 (SE: 84.8) per 10,000 deliveries), New Hampshire (3,376 (SE: 100.9) per 10,000 deliveries), and Vermont (3,273 (SE: 119.5) per 10,000 deliveries), exhibited the highest PMAD diagnosis rates in 2021, with Maine and Vermont experiencing the highest PMAD diagnosis rates in both 2017 and 2021. Figure 2 values and standard errors can be found in Supplementary Table S2.

The only state to exhibit a decrease in PMAD diagnosis rates per 10,000 deliveries was Georgia (-12.57% relative decrease; -1.40% absolute decrease), from 1,115 (SE: 22.9) to 974 (SE: 16.6) per 10,000 deliveries. Massachusetts, which had one of the highest PMAD diagnosis rates in 2017, reflected the smallest increase (3.06% relative increase; 0.70% absolute increase), from 2,276 (SE: 30.9) to 2,345 (SE: 33.0) per 10,000 deliveries. Tennessee and Maryland exhibited the largest increases in PMAD diagnosis rates for which these rates more than doubled from 2017 to 2021. Tennessee experienced a 107% relative increase (8.59% absolute increase), from 803 (SE: 16.9) to 1,662 (SE: 25.0) per 10,000 deliveries. Maryland experienced a 136.94% relative increase (11.79% absolute increase), from 861 (SE: 21.3) to 2,039 (SE: 30.8) per 10,000 deliveries.

**Fig. 3:**
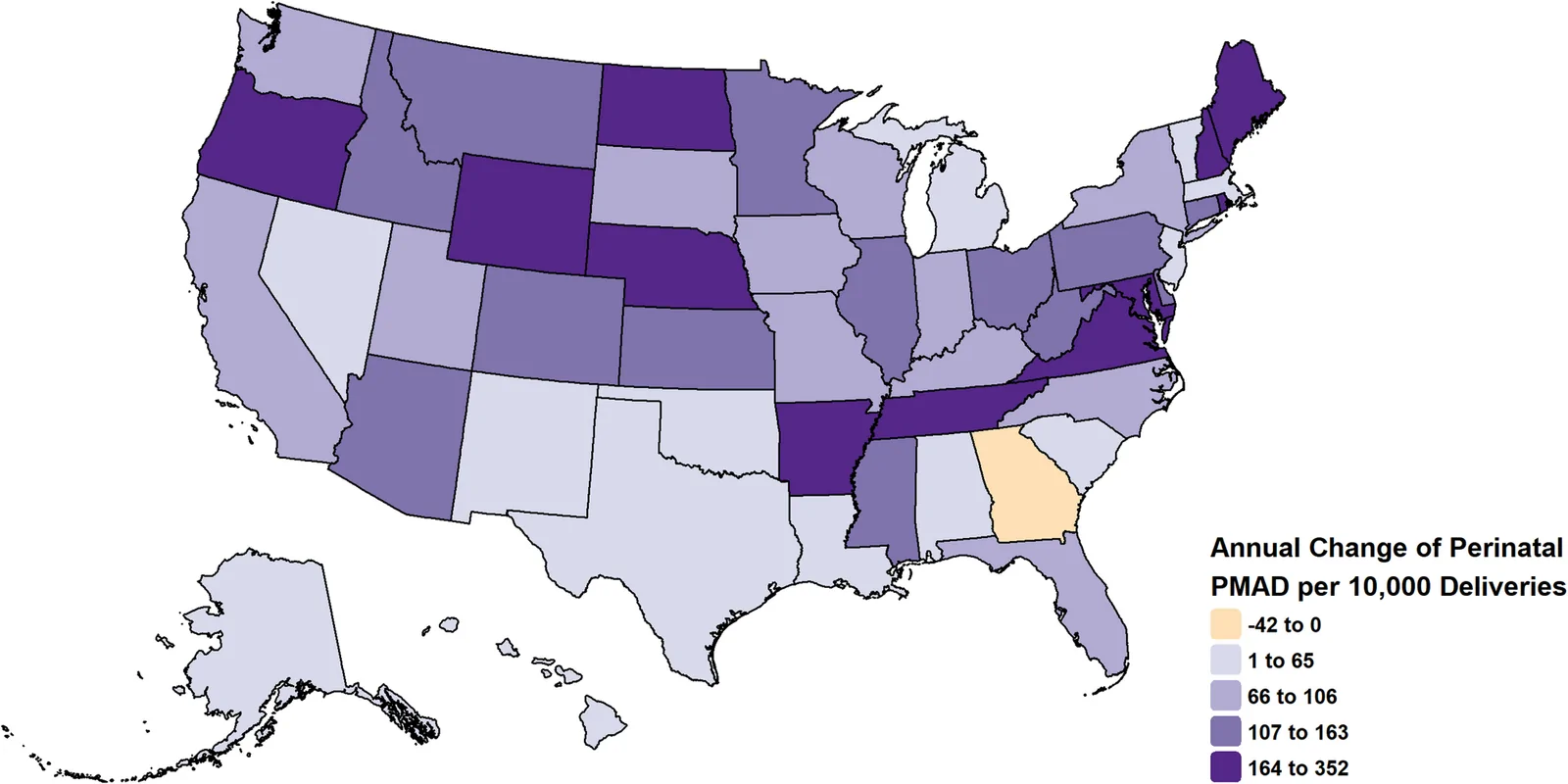
Annual rate of change in the number of diagnosed perinatal mood and anxiety disorders (PMAD) per 10,000 deliveries in the U.S. among Medicaid insured women, 2017–2021. *Mississippi’s annual change is based off of 2017–2020, 2021 is suppressed due to low counts.

Figure 3 shows the annual rate of change in PMAD diagnoses per 10,000 deliveries during 2017–2021, by state. Mean annual change across all states reached 120 additional PMAD diagnoses per 10,000 deliveries (95% CI: 68 to 171) (Supplementary Table S3). Of the 50 states including Washington, D.C. with a positive point estimate, 38 states experienced a statistically significant increase. New Hampshire exhibited the greatest annual growth, accruing an average increase of 352 PMAD diagnoses per 10,000 deliveries annually (95% CI: 186 to 519). Georgia experienced the only annual decrease g of -42 per 10,000 deliveries per year (95% CI: -70 to − 15). All state-specific state annual change values appear in Supplementary Table S3.

In regional stratification (Supplementary Table S3), the South had the highest average annual change in PMAD diagnoses per 10,000 deliveries(122 per 10,000 deliveries, 95% CI: 67 to 177), followed by the Midwest (112 per 10,000 deliveries, 95% CI: 49 to 175), and West (107 per 10,000 deliveries, 95% CI: 7 to 208). Although the Northeast had the highest magnitude, we found no statistically significant change over time (136 per 10,000 deliveries, 95% CI: -28 to 301).

**Fig. 4:**
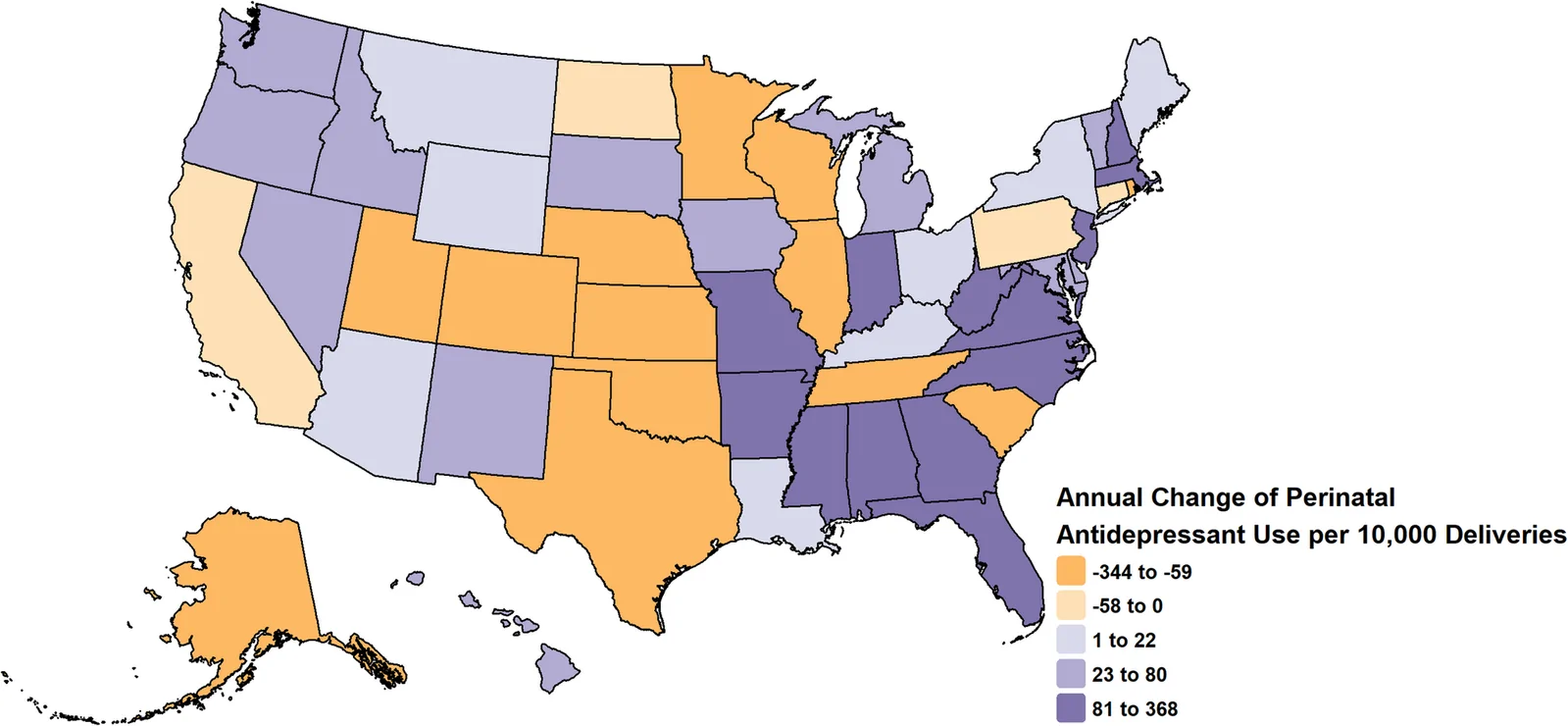
Annual rate of change in the number of those with any antidepressant prescription among women with diagnosed perinatal mood and anxiety disorders (PMAD) per 10,000 deliveries in the U.S. among Medicaid insured women, 2017–2021. *Mississippi’s annual change is based off of 2017–2020, 2021 is suppressed due to low counts.

Figure 4 shows the annual rate of change in antidepressant prescriptions per 10,000 deliveries among those with PMAD diagnoses during 2017–2021 by state. Taken together, antidepressant prescriptions across the country remained flat at ~ 46% among those with PMAD in all years, with a mean annual change of 15 additional individuals using antidepressants per 10,000 deliveries across all states (95% CI: -70 to 102) (Supplementary Table S3). However, as illustrated in the U.S. map, substantial variation in antidepressant prescriptions occurred across states. Point estimates were negative for 17 states and positive for 34 states including Washington, D.C. Of these, five states (Florida, Georgia, Massachusetts, North Carolina, and West Virgina) showed statistically significant increases, and six states (Kansas, Oklahoma, Rhode Island, South Carolina, Texas, and Wisconsin) showed statistically significant decreases. North Carolina exhibited the largest annual growth of 368 per 10,000 deliveries (95% CI: 133 to 603). Rhode Island experienced the largest decrease in antidepressant use of -344 per 10,000 deliveries (95% CI: -505 to -182). All state-specific annual change values appear in Supplementary Table S3.

In the regional stratification (Supplementary Table S3), we found no statistically significant annual rates of change. The South had the largest average annual change in antidepressant use among those with PMAD, with 52 per 10,000 deliveries (95% CI: -122 to 226), followed by the Northeast (7 per 10,000 deliveries, 95% CI: -113 to 126), and the West (1 per 10,000 deliveries, 95% CI: -168 to 170). The Midwest experienced a negative annual change of  -16 per 10,000 deliveries (95% CI: -100 to 69).

**Fig. 5:**
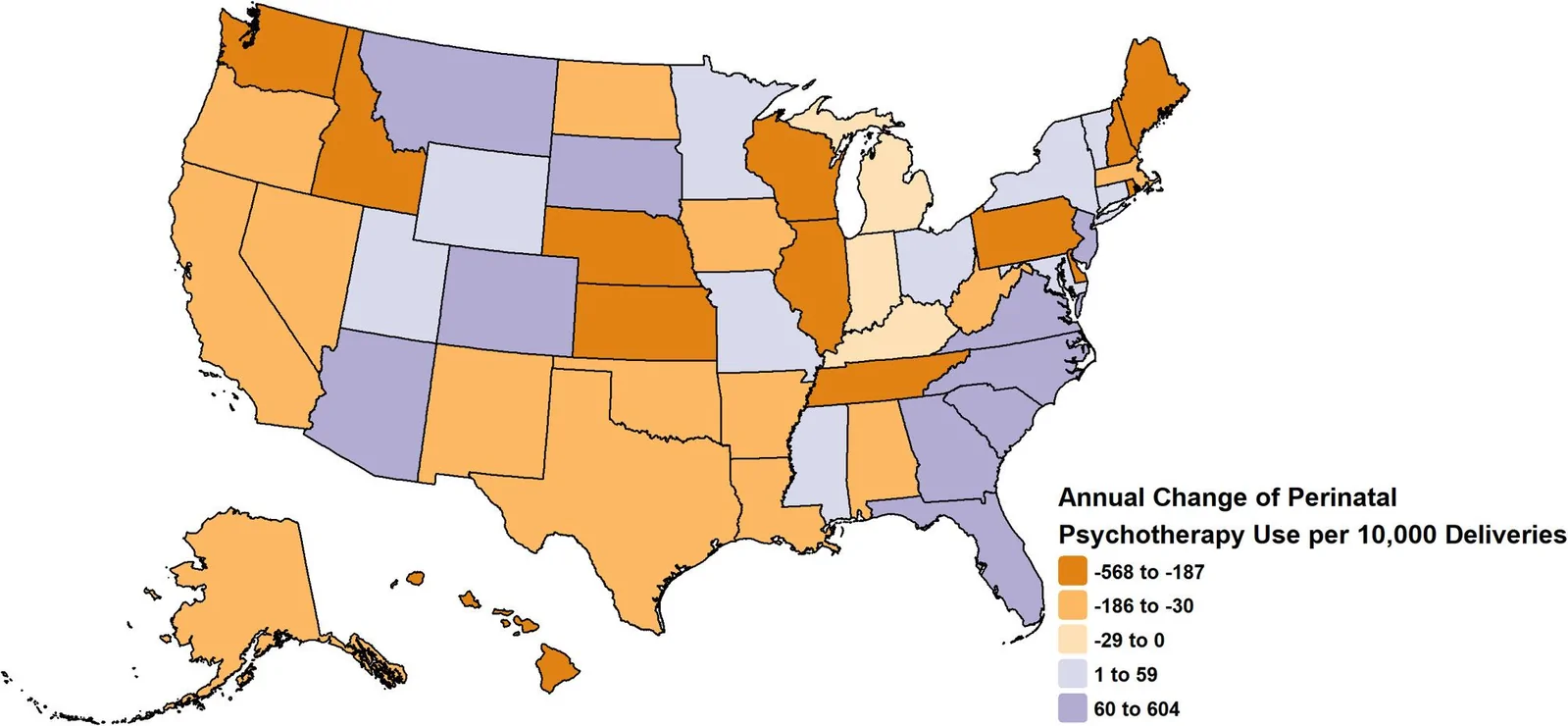
Annual rate of change in the number of those with any psychotherapy use among women with diagnosed perinatal mood and anxiety disorders (PMAD) per 10,000 deliveries in the U.S. among Medicaid insured women, 2017–2021. *Mississippi’s annual change is based off of 2017–2020, 2021 is suppressed due to low counts.

Figure 5 shows the annual rate of change in psychotherapy use per 10,000 deliveries among those with PMAD diagnoses during 2017–2021 by state. Taken together, psychotherapy use declined somewhat across the country from 4,861 per 10,000 deliveries in 2017 to 4,544 per 10,000 deliveries in 2021 among those with PMAD diagnoses, with an average annual change of -61 per 10,000 deliveries (95% CI: -169 to 47) (Supplementary Table S3). However, as illustrated in the U.S. map, substantial variation in psychotherapy use occurred across states, with 30 states experiencing negative and 21 states including Washington, D.C. experiencing positive point estimates over the five-year period. Of these, five states (Colorado, Florida, Georgia, New Jersey, and Virgina) demonstrated a statistically significant increase, and 14 states (Alabama, Delaware, Hawaii, Iowa, Illinois, Kansa, Louisiana, Maine, Oklahoma, Pennsylvania, Rhode Island, Tennessee, Washington, and Wisconsin) demonstrated a statistically significant decrease. Colorado exhibited the largest annual growth of 604 per 10,000 deliveries (95% CI: 354 to 854) and Tennessee experienced the largest decline of  -568 per 10,000 deliveries per year (95% CI: -972 to -165). All state-specific annual change values appear in Supplementary Table S3.

In the regional stratification (Supplementary Table S3), we found no statistically significant annual rates of change. The Northeast experiences the largest absolute annual change (-143 per 10,000 deliveries, 95% CI: -484 to 199), followed by the Midwest (-82 per 10,000 deliveries, 95% CI: -195 to 32), the West (-51 per 10,000 deliveries, 95% CI: -253 to 150), and the South (-14 per 10,000 deliveries, 95% CI: -198 to 169).

## Discussion

This study sought to characterize the prevalence, diagnosis, and treatment rates for PMAD among women with Medicaid health insurance coverage in the U.S. from 2017 to 2021. We found that PMAD diagnosis rates increased over a five-year period in Medicaid across most states and subgroups. Across all race and ethnic subgroups, the greatest number of diagnoses existed for White women, but non-Hispanic Multiracial women experienced the largest increase in diagnoses. Among age groups, younger women (ages 20–24) experienced the greatest increase. Our study also characterizes PMAD diagnosis rates in some population subgroups previously not characterized in other studies, such as in AIAN women. We found substantial geographic variation in treatment rates.

These results align with previous findings in a commercially insured sample. Our team found that PMADs rose among privately insured women from 2008 to 2020^5^. In the current study, women with Medicaid insurance had lower PMAD diagnosis rates compared to women with private insurance in our previous study. We restricted cohorts for both studies to women continually enrolled in Medicaid and commercial insurance during the perinatal period; however, important differences between populations who continuously enroll in either insurance type may exist, making these groups difficult to compare. Although the commercially insured in many states had increases in psychotherapy rates, as in the present study, we found substantial variation and some decreases over time^5^. Our prior work in a commercially insured population did not focus on state variation, but indicated increased use in antidepressants nationwide between 2017 and 2020^22^.

Multiracial women enrolled in Medicaid experienced the greatest increase in PMAD irrespective of the state rates. This finding is remarkable and aligns with several recent investigations on perinatal mental health outcomes comparing single-race and multi-race women. A recent report of the prevalence of bipolar disorder in the state of California^40^ found an increased burden of bipolar diagnosis among Multiracial women. Another study using surveillance data from the Pregnancy Risk Assessment Monitoring System (PRAMS) found that Multiracial respondents experienced more postpartum depressive symptoms, diagnosis, and treatment compared to other race and ethnicity groups^41^. The results from the present study align with growing curiosity^42^ about the existence of perinatal mental health conditions among Multiracial women and highlights the need to identify appropriate and tailored clinical solutions^43^.

Women with Medicaid may exhibit more mental health risk factors and higher risk for mental health conditions than women with commercial insurance^44^ while facing greater barriers to accessing mental health care such as cost and transportation^45,46^. Additionally, women with Medicaid may attend fewer visits due to barriers to accessing routine obstetric care^47^ leading to fewer opportunities to detect PMAD, although we did not assess how many visits women attended in either type of coverage in either of our studies. Nevertheless, similar data patterns emerged in both studies, with overall increases in PMAD across the majority of states and subgroups with substantial geographic variability.

Increases in PMAD diagnoses may reflect several interrelated factors. The rise in diagnoses may reflect growing patient and provider awareness of illness, especially after 2016 Medicaid policies to increase screening^48^ and 2015 American College of Obstetricians and Gynecologists guidelines for screening for perinatal depression^49^. Additionally, increased diagnoses may reflect overall growth of awareness about U.S. maternal morbidity and mortality, both at the policy level^50^ and in popular media. Finally, increases in PMAD diagnoses may reflect a true rise in disease prevalence.

Treatment rates have a relationship with the presence of a diagnosis when adequate referral and follow-up occur. The increase in diagnosis rates may therefore partially explain the more modest, sometimes decreasing, changes in treatment rates. Improvements in detection may concentrate on mild cases that were previously missed, and patients with milder PMAD may warrant or prefer fewer treatment modalities. However, claims cannot assess symptom severity, so we cannot account for characteristics of PMAD, patient preference, or history of mental health conditions or prior treatment.

Characterizing rates of PMAD diagnoses and treatment represents an important first step. However, future research should aim to better understand factors underlying variation in PMAD diagnoses and treatment rate across subgroups and geographic areas. Community characteristics such as rural or urban status^51^ and related differential access to healthcare providers may play an important role.

Additionally, researchers could assess the impact of inter-state variation in perinatal programs and resources on PMAD diagnoses. For example, many states offer perinatal psychiatry access programs, which aim to increase access to PMAD treatment by offering free, same-day, telephone psychiatric consultations to providers who treat pregnant and postpartum women with mental health concerns^52^, but other states do not. Whether states reimburse for screening for postpartum depression at infant well-child visits represents another source of inter-state variation^53^. Differences in state-level requirements for mandated depression screening and to what extent Medicaid covers psychotherapy could impact the ability to detect individuals in need of PMAD diagnosis and treatment rates^54–57^. Several states have mandated depression screening during pregnancy and postpartum, but few states have evaluated the implementation and effectiveness of policy mandates to improve perinatal mental health.

### Strengths and limitations

This study has several important strengths. Compared to previous studies of PMAD in Medicaid births, our study analyzed a large, national, and recent dataset representing millions of births. We also examined patient level variation in diagnosis and treatment over time.

This study also has limitations. Our study period (2017–2021) included the beginning of the COVID-19 pandemic, a period when the pandemic potentially impacted health care use, due to lockdown policies and Medicaid ceased disenrollment potentially altering study population composition. Our sample included women continuously enrolled in Medicaid for 12 months, representing a population not generalizable to those experiencing gaps in coverage. We did not evaluate risk factors beyond nine months pre-delivery such as history of pre-pregnancy mental health conditions or other co-morbidities. Further, we did not evaluate outcomes beyond three months post-delivery due to limits to Medicaid coverage beyond 60 days among those who qualified for coverage based on pregnancy status, even though PMAD can occur up to one year postpartum. The American Rescue Plan Act of 2021 gave states the option to extend Medicaid coverage from 60 days to 12 months post-delivery; findings in this analysis do not incorporate a full year post-delivery given that states did not implement postpartum extensions until after our study period (i.e., most states implemented the extensions in 2022 and 2023)^58^. Additionally, Medicaid benefits vary across states, including for covered services for prenatal care and mental health; this variation is a potential source of heterogeneity in diagnosis and treatment rates.

We also note that the underlying increase in individuals reporting their race and ethnicity as multiracial may in part have to do with more category choices included in Medicaid enrollment, which could also then explain decreases for those in the other racial subgroups and may have influenced findings^59^. Additionally, researchers have documented variation in PMAD diagnoses among different racial and ethnic groups which may stem from complex structural or cultural factors^41,60^. Finally, analyses excluded non-live births, yet women undergoing pregnancy loss could experience PMAD.

## Conclusions

Although PMAD diagnosis nationwide and across population subgroups largely increased over time, factors not observable in Medicaid administrative data might help further explain substantial variation in treatment rates. Future work may consider community-level variables ranging from socioeconomic conditions such as poverty and unemployment rates, to availability of maternity care and mental health providers, as many individuals nationwide live in health care provider shortage areas^61,62^. Understanding not only risk factors for diagnosis but conditions associated with potential gaps in treatment represents an important next step in filling gaps and addressing unmet needs among pregnant women and new mothers in the US and worldwide.

## Supplementary Information

Below is the link to the electronic supplementary material.

Supplementary Material 1

## Data Availability

The data that support the findings of this study are available from the Centers for Medicare & Medicaid Services (CMS) but restrictions apply to the availability of these data, which were used under license for the current study, and so are not publicly available. Data are however available from the authors upon reasonable request to the corresponding author (KZ) and with permission of CMS.
