# Supplementary material for: Perinatal mood and anxiety diagnoses and treatment increased among medicaid births, 2017–2021
Source: Sci Rep. 2026 Jun 18;16:27771. doi: 10.1038/s41598-026-57879-3 (PMC13542285; doi:10.1038/s41598-026-57879-3)
Supplement: Supplementary file 1 [file 41598_2026_57879_MOESM1_ESM.docx]

###### **Supplementary Table S1: Codes used to create delivery cohort, perinatal mood and anxiety disorder (PMAD) diagnoses, antidepressant prescriptions, psychotherapy visits**

Deliveries

*Included: Cesarean and vaginal delivery*

|  | Cesarean delivery | Vaginal delivery |
| --- | --- | --- |
| ICD-10-CM procedures | 10D00Z0, 10D00Z1, 10D00Z2 | 10D07Z3, 10D07Z4, 10D07Z5, 10D07Z6, 10D07Z7, 10D07Z8, 10E0ZZ |
| DRG | 370, 371, 765, 766, 540, 5401, 5402, 5403, 5404 | 372, 373, 374, 375, 767 768, 774, 775, 541, 542, 560, 5411, 5412, 5413, 5414, 5421, 5422, 5423, 5424, 5601, 5602, 5603 |
| CPT | 59510, 59514, 59515, 59618, 59620, 59622 | 59400, 59409, 59410, 59610, 59612, 59614 |
|  |  |  |

*Included: Pre-term birth*

| ICD 10 diagnoses | O601, O6010, O60100, O60101, O60102, O60103, O60104, O60105, O60109, O6012, O60120, O60121, O60122, O60123, O60124, O60125, O60129, O6013, O60130, O60131, O60132, O60133, O60134, O60135, O60139, O6014, O60140, O60141, O60142, O60143, O60144, O60145, O60149 |
| --- | --- |

*Excluded: abortion, dilatation and curettage, and stillbirth*

| ICD procedures and CPT codes | Removal of ectopic pregnancy | 59120, 59121, 59130, 59135, 59136, 59140, 59150, 59151, 743 |
| --- | --- | --- |
|  | Abortion (termination of pregnancy) | 59840, 59841, 59850, 59851, 59852, 59855, 59856, 59857, 10A00ZZ, 10A03ZZ, 10A04ZZ, 10A07Z, 10A07ZZ, 10A07ZZ, 10A08ZZ, 10A08ZZ, 6901, 6951, 7491, 750 |
|  | Dilatation and curettage (D&C); aspiration after delivery or abortion | 59812, 59820, 59821, 59830 |

| ICD 10 diagnoses | Removal of ectopic pregnancy | O0000, O0001, O00101, O00102, O00109, O00111, O00112, O00119, O00201, O00202, O00209, O00211, O00212, O00219, O0080, O0081, O0090, O0091 |
| --- | --- | --- |
|  | Abortion (termination of pregnancy) | O030, O031, O032, O0330, O0331, O0332, O0333, O0334, O0337, O0339, O034, O035, O036, O037, O0380, O0381, O0382, O0383, O0384, O0385, O0386, O0387, O0388, O0389, O039, O045, O045, O045, O046, O046, O046, O047, O047, O047, O0480, O0480, O0480, O0481, O0481, O0481, O0482, O0482, O0482, O0483, O0483, O0483, O0484, O0484, O0484, O0485, O0486, O0487, O0488, O0489, O0489, O0489, O070, O071, O072, O0730, O0731, O0732, O0733, O0734, O0735, O0736, O0737, O0738, O0739, O074,O080, O081, O082, O083, O084, O085, O086, O087, O0881, O0882, O0883, O0889, O089 |
|  | Other complications | O019, O021, O0289,A34 |

Bateman Comorbidity Index (OBCMI)

| ICD 10 diagnosis codes | F10, J44, J45, I05, I06, I07, I08, I09, I34, I35, I36, I37, I38, I39, I500, I20, I25, N022,N03, N04, N05, N08, N171,N172,N18, N25, O268,Q20, Q21, Q22, Q23, Q24, Q25, Q26, O994, F11, F12, F13, F14, F15, F16, F18, F19, O13, O16, B20, B24, O987, Z21, O11, O14, O30, O31, Z372,Z373, Z374, Z375,Z376,Z377,Z3790, O44, E10, E11, O245, O246,O247,I10, I11, I12, I13, I15, O10, O11, O3420, I270,I272, I278,I279, O14, O15, D56, D57, M32 |
| --- | --- |

PMAD

| Group | ICD Diagnosis Code |
| --- | --- |
| Anxiety | F06, F40, F41, F42, F43, F48, R45 |
| Depression | F32, F33 |

Antidepressants

| HIC3 | H24, H2H, H2J, H2K, H2N, H2S, H2U, H2Y, H7B, H7C, H7D, H7E, H7I, H7J, H7K, H7L, H8P, H8S, H8T, H8Z |
| --- | --- |

Psychotherapy visits

| CPT/HCPCS | 90804, 90805, 90806, 90807, 90808, 90809, 90810, 90811, 90812, 90813, 90814, 90815, 90816, 90817, 90818, 90819, 90820, 90821, 90822, 90823, 90824, 90826, 90827, 90828, 90829, 90832, 90833, 90834, 90836, 90837, 90838, 90839, 90840, 90841, 90842, 90843, 90844, 90846, 90847, 90849, 90853, 90855 |
| --- | --- |

###### **Supplementary Table S2: PMAD diagnoses per 10,000 deliveries and standard errors in 2017 and 2021 by demographic and clinical characteristics, and state**

|  | **PMAD Diagnoses per 10,000 Deliveries (SE)** | |
| --- | --- | --- |
|  | **2017** | **2021** |
| **Age** |  |  |
| *15-19* | 1,145 (11.1) | 1,434 (13.8) |
| *20-24* | 1,052 (5.8) | 1,463 (6.9) |
| *25-29* | 1,185 (5.9) | 1,526 (6.6) |
| *30-35* | 1,301 (7.2) | 1,551 (7.2) |
| *36+* | 1,301 (11.4) | 1,523 (11.2) |
| **Race and Ethnicity** |  |  |
| *American Indian and Alaska Native (AIAN), non-Hispanic* | 1,355 (32.7) | 1,683 (33.5) |
| *Asian, non-Hispanic* | 356 (14.0) | 561 (17.2) |
| *Black, non-Hispanic* | 870 (7.2) | 1,119 (7.2) |
| *Native Hawaiian/Pacific Islander* | 556 (37.4) | 726 (45.0) |
| *Hispanic, all races* | 675 (6.2) | 939 (6.6) |
| *Multiracial, non-Hispanic* | 1,319 (65.8) | 1,856 (43.7) |
| *Other, non-Hispanic* | 1,093 (19.8) | 1,319 (22.3) |
| *White, non-Hispanic* | 1,707 (7.6) | 2,132 (7.5) |
| **Region** |  |  |
| *Midwest* | 1,524 (9.1) | 1,801 (9.7) |
| *Northeast* | 1,530 (7.8) | 1,890 (8.7) |
| *South* | 980 (5.1) | 1,309 (5.4) |
| *West* | 944 (5.7) | 1,317 (7.0) |
| **OBCMI** |  |  |
| *0-1* | 1,037 (3.4) | 1,361(3.8) |
| *2+* | 1,958 (10.3) | 2,266 (10.4) |
| **State** |  |  |
| *Alaska* | 1,310 (62.8) | 1,468 (65.3) |
| *Alabama* | 954 (25.8) | 1,165 (23.0) |
| *Arkansas* | 793 (32.9) | 1,520 (41.9) |
| *Arizona* | 863 (14.9) | 1,373 (20.9) |
| *California* | 633 (6.8) | 998 (8.8) |
| *Colorado* | 1,192 (25.2) | 1,762 (30.8) |
| *Connecticut* | 1,735 (38.4) | 2,163 (40.9) |
| *District of Columbia* | 1,125 (55.7) | 1,876 (73.7) |
| *Delaware* | 1,279 (59.3) | 1,695 (70.1) |
| *Florida* | 602 (8.9) | 909 (10.5) |
| *Georgia* | 1,115 (22.9) | 974 (16.6) |
| *Hawaii* | 615 (34.2) | 688 (40.4) |
| *Iowa* | 1,918 (37.2) | 2,171 (40.5) |
| *Idaho* | 1,987 (74.8) | 2,170 (59.6) |
| *Illinois* | 1,127 (15.5) | 1,559 (19.7) |
| *Indiana* | 1,136 (20.7) | 1,434 (21.7) |
| *Kansas* | 1,660 (50.0) | 2,109 (54.9) |
| *Kentucky* | 1,486 (23.2) | 1,857 (24.9) |
| *Louisiana* | 1,102 (20.4) | 1,318 (20.8) |
| *Massachusetts* | 2,276 (30.9) | 2,345 (33.0) |
| *Maryland* | 861 (21.3) | 2,039 (30.8) |
| *Maine* | 2,865 (86.9) | 3,589 (84.8) |
| *Michigan* | 1,497 (19.2) | 1,750 (20.5) |
| *Minnesota* | 1,953 (31.4) | 2,400 (36.2) |
| *Missouri* | 1,226 (29.0) | 1,433 (31.4) |
| *Mississippi* | 740 (25.4) | 1,116 (214.8) |
| *Montana* | 2,035 (70.7) | 2,650 (77.1) |
| *North Carolina* | 1,321 (19.1) | 1,661 (20.4) |
| *North Dakota* | 1,629 (95.0) | 2,232 (109.5) |
| *Nebraska* | 1,271 (76.5) | 2,032 (61.3) |
| *New Hampshire* | 1,994 (95.8) | 3,376 (100.9) |
| *New Jersey* | 646 (19.4) | 713 (18.8) |
| *New Mexico* | 1,119 (28.6) | 1,255 (31.8) |
| *Nevada* | 1,025 (31.8) | 1,074 (32.1) |
| *New York* | 1,184 (12.4) | 1,442 (13.7) |
| *Ohio* | 1,889 (17.8) | 2,358 (20.6) |
| *Oklahoma* | 1,652 (39.9) | 1,721 (35.6) |
| *Oregon* | 1,626 (30.6) | 2,173 (37.4) |
| *Pennsylvania* | 1,936 (21.4) | 2,306 (22.0) |
| *Rhode Island* | 1,487 (61.6) | 2,288 (76.4) |
| *South Carolina* | 903 (19.5) | 1,068 (21.4) |
| *South Dakota* | 1,529 (96.4) | 1,824 (98.9) |
| *Tennessee* | 803 (16.9) | 1,662 (25.0) |
| *Texas* | 912 (16.0) | 1,015 (11.5) |
| *Utah* | 2,061 (54.5) | 2,318 (63.2) |
| *Virginia* | 1,107 (24.6) | 1,722 (25.9) |
| *Vermont* | 3,015 (105.1) | 3,273 (119.5) |
| *Washington* | 1,546 (24.4) | 1,794 (27.0) |
| *Wisconsin* | 1,677 (30.0) | 1,988 (31.2) |
| *West Virginia* | 1,543 (40.9) | 2,067 (48.1) |
| *Wyoming* | 1,342 (78.8) | 2,408 (158.1) |

###### **Supplementary Table S3: Annual change estimate and 95% confidence interval overall, by region and by state for perinatal mood and anxiety disorder (PMAD) diagnoses, antidepressant prescriptions and psychotherapy use amongst those with PMAD from 2017-2021**

|  | **PMAD** | | **Antidepressant use amongst PMAD** | | **Psychotherapy use**  **amongst PMAD** | |
| --- | --- | --- | --- | --- | --- | --- |
|  | Annual change estimate | 95% CI | Annual change estimate | 95% CI | Annual change estimate | 95% CI |
| **Overall** | 119.67 | **68.47, 170.87** | 15.96 | -70.03, 101.95 | -61.03 | -169.36, 47.30 |
| **Region** |  |  |  |  |  |  |
| *Midwest* | 112.40 | **49.37, 175.44** | -15.57 | -100.23, 69.10 | -81.81 | -195.41, 31.80 |
| *Northeast* | 136.27 | -28.38, 300.92 | 6.61 | -113.17, 126.40 | -142.60 | -483.97, 198.77 |
| *South* | 121.79 | **66.98, 176.59** | 51.68 | -122.14, 225.50 | -14.16 | -197.73, 169.40 |
| *West* | 107.43 | **7.00, 207.86** | 1.16 | -167.68, 170.00 | -51.46 | -252.91, 149.99 |
| **State** |  |  |  |  |  |  |
| *Alaska* | 64.07 | **4.18, 123.95** | -105.47 | -211.93, 0.98 | -77.79 | -250.26, 94.68 |
| *Alabama* | 58.92 | **35.72, 82.11** | 87.69 | -37.37, 212.75 | -144.69 | **-243.52, -45.85** |
| *Arkansas* | 181.46 | **83.63, 279.30** | 158.57 | -146.78, 463.92 | -162.77 | -357.81, 32.26 |
| *Arizona* | 136.99 | **96.91, 177.08** | 17.81 | -66.83, 102.45 | 91.07 | -76.26, 258.40 |
| *California* | 98.61 | **68.33, 128.90** | -24.69 | -144.77, 95.39 | -173.18 | -412.53, 66.16 |
| *Colorado* | 163.09 | **82.95, 243.23** | -61.23 | -165.38, 42.92 | 604.06 | 353.97, 854.16 |
| *Connecticut* | 133.28 | **59.63, 206.93** | -47.52 | -156.81, 61.76 | 28.28 | -182.51, 239.08 |
| *District of Columbia* | 209.39 | **153.77, 265.01** | 9.39 | -59.31, 78.08 | 3.86 | -85.81, 93.53 |
| *Delaware* | 145.48 | **47.88, 243.09** | 71.97 | -18.51, 162.46 | -234.22 | **-408.78, -59.65** |
| *Florida* | 91.46 | **57.51, 125.41** | 193.13 | **15.71, 370.54** | 528.28 | **45.88, 1010.68** |
| *Georgia* | -42.47 | **-69.64, -15.30** | 151.24 | **6.57, 295.92** | 101.58 | **26.25, 176.91** |
| *Hawaii* | 16.61 | -10.46, 43.68 | 39.24 | -87.14, 165.61 | -476.88 | **-849.41, -104.36** |
| *Iowa* | 74.45 | -39.41, 188.31 | 28.50 | -76.99, 134.00 | -170.91 | **-225.43, -116.39** |
| *Idaho* | 107.71 | -90.83, 306.25 | 32.76 | -164.80, 230.32 | -312.85 | -985.62, 359.91 |
| *Illinois* | 124.63 | **69.39, 179.86** | -87.74 | -210.08, 34.61 | -407.57 | **-615.75, -199.40** |
| *Indiana* | 77.30 | **62.28, 92.33** | 81.35 | -63.46, 226.15 | -24.76 | -72.45, 22.92 |
| *Kansas* | 116.42 | **21.22, 211.62** | -62.88 | **-118.98, -6.78** | -231.35 | **-333.83, -128.87** |
| *Kentucky* | 105.84 | **50.59, 161.08** | 13.60 | -81.34, 108.55 | -24.48 | -113.03, 64.06 |
| *Louisiana* | 52.51 | -4.35, 109.37 | 3.69 | -90.59, 97.97 | -102.54 | **-182.58, -22.51** |
| *Massachusetts* | 4.47 | -50.02, 58.96 | 143.17 | **15.99, 270.34** | -118.10 | -282.32, 46.12 |
| *Maryland* | 274.90 | **64.55, 485.26** | 31.10 | -90.55, 152.74 | 51.58 | -195.25, 298.40 |
| *Maine* | 249.74 | **64.13, 435.34** | 5.06 | -192.93, 203.06 | -313.81 | **-583.81, -43.80** |
| *Michigan* | 63.59 | **46.91, 80.27** | 65.59 | -17.48, 148.66 | -5.83 | -122.64, 110.98 |
| *Minnesota* | 124.28 | **80.40, 168.15** | -63.69 | -134.26, 6.88 | 54.37 | -79.77, 188.52 |
| *Missouri* | 69.41 | **17.68, 121.14** | 114.04 | -19.28, 247.36 | 29.45 | -233.60, 292.51 |
| *Mississippi* | 131.30 | **83.14, 179.45** | 125.66 | -340.84, 592.15 | 37.30 | -42.52, 117.12 |
| *Montana* | 160.14 | **79.99, 240.30** | 22.22 | -137.99, 182.43 | 97.54 | -87.65, 282.73 |
| *North Carolina* | 97.08 | **65.23, 128.94** | 367.77 | **132.85, 602.70** | 146.67 | -88.02, 381.37 |
| *North Dakota* | 167.52 | **97.10, 237.94** | -57.40 | -193.22, 78.41 | -30.65 | -218.04, 156.73 |
| *Nebraska* | 197.08 | **142.31, 251.85** | -138.75 | -317.57, 40.06 | -202.11 | -443.28, 39.07 |
| *New Hampshire* | 352.27 | **185.57, 518.96** | 119.45 | -16.63, 255.54 | -222.96 | -557.56, 111.65 |
| *New Jersey* | 23.51 | -3.14, 50.15 | 104.09 | -0.25, 208.42 | 87.97 | **15.96, 159.99** |
| *New Mexico* | 50.66 | -19.43, 120.75 | 34.34 | -89.14, 157.82 | -107.50 | -272.42, 57.43 |
| *Nevada* | 21.94 | -28.98, 72.86 | 54.07 | -75.68, 183.83 | -30.51 | -200.89, 139.87 |
| *New York* | 80.91 | **23.77, 138.05** | 11.77 | -104.75, 128.29 | 28.95 | -116.90, 174.80 |
| *Ohio* | 152.47 | **50.69, 254.24** | 3.82 | -122.54, 130.18 | 14.85 | -158.14, 187.83 |
| *Oklahoma* | 40.08 | -59.64, 139.79 | -160.50 | **-318.63, -2.37** | -106.81 | **-158.76, -54.85** |
| *Oregon* | 164.75 | **79.71, 249.79** | 52.20 | -104.97, 209.36 | -33.65 | -256.02, 188.73 |
| *Pennsylvania* | 109.62 | **33.06, 186.18** | -13.48 | -103.62, 76.65 | -274.93 | **-375.85, -174.02** |
| *Rhode Island* | 231.38 | **123.51, 339.25** | -343.53 | **-504.70, -182.36** | -521.71 | **-920.41, -123.02** |
| *South Carolina* | 46.49 | **29.04, 63.95** | -187.68 | **-349.89, -25.47** | 60.95 | -1.55, 123.45 |
| *South Dakota* | 102.82 | **18.22, 187.41** | 65.45 | -83.38, 214.27 | 205.49 | -24.73, 435.71 |
| *Tennessee* | 244.87 | **126.30, 363.45** | -119.68 | -340.53, 101.18 | -568.11 | **-971.54, -164.67** |
| *Texas* | 38.76 | -26.41, 103.94 | -61.10 | -274.13, 151.93 | -135.62 | -280.91, 9.67 |
| *Utah* | 97.39 | -94.58, 289.36 | -92.13 | -273.91, 89.64 | 2.57 | -217.87, 223.01 |
| *Virginia* | 168.40 | **123.96, 212.85** | 103.26 | -36.04, 242.57 | 347.83 | **100.53, 595.13** |
| *Vermont* | 41.26 | -32.70, 115.22 | 80.52 | -73.21, 234.25 | 22.89 | -417.21, 462.99 |
| *Washington* | 68.40 | **42.67, 94.12** | 35.93 | -156.59, 228.46 | -311.62 | **-604.26, -18.98** |
| *Wisconsin* | 78.87 | **39.82, 117.91** | -135.08 | **-168.82, -101.33** | -212.65 | **-297.07, -128.23** |
| *West Virgina* | 161.12 | **72.47, 249.76** | 161.27 | **52.32, 270.21** | -125.17 | -514.02, 263.68 |
| *Wyoming* | 246.19 | **141.51, 350.87** | 10.06 | -247.90, 268.02 | 59.78 | -132.54, 252.10 |

*Mississippi’s annual change is modeled without 2021 due to low counts for all outcomes. Colors correspond to the legend for each outcome’s annual change map. Bold 95% CI indicate statistical significance at the 0.05 alpha level.

**Supplementary Table S3: States with medium to low concern race and ethnicity data quality included in race and ethnicity descriptive statistics**
